# Recent advances in research on the multifactorial mechanisms underlying paclitaxel resistance and translational intervention strategies

**DOI:** 10.3389/fphar.2026.1909497

**Published:** 2026-09-11

**Authors:** Zhu Mingjun, Zhou Bingwen, Wang Lu, Fan Zhimin

**Affiliations:** 1 Nanjing University of Chinese Medicine, Nanjing, Jiangsu, China; 2 Nanjing Hospital of Chinese Medicine affiliated to Nanjing University of Chinese Medicine, Clinical Innovation Center For Anorectal Diseases, Nanjing, Jiangsu, China

**Keywords:** ABC transporters, autophagy, drug resistance, microtubule remodeling, paclitaxel, tumor microenvironment

## Abstract

Paclitaxel (PTX) is a well-established chemotherapeutic agent that is widely used in clinical practice. It exerts its antitumor activity primarily by stabilizing microtubules and disrupting mitosis, and it is currently used in the treatment of various solid tumors, including ovarian, breast, non-small cell lung, and gastric cancer. However, intrinsic and acquired resistance in tumor cells significantly limit the clinical efficacy of PTX and are closely associated with treatment failure, tumor recurrence, and poor prognosis. PTX resistance is not driven by a single mechanism but instead arises from the combined effects of multiple regulatory processes, including enhanced drug efflux, microtubule remodeling, evasion of apoptosis, dysregulated autophagic homeostasis, adaptation of the tumor microenvironment (TME), metabolic reprogramming, and aberrant epigenetic regulation. This review systematically summarizes the major molecular mechanisms underlying PTX resistance, with a particular focus on the dynamic interactions among distinct resistance pathways and their regulatory network architecture. It also outlines current resistance-overcoming strategies based on nanodelivery systems, combination therapies, and interventions targeting emerging molecular determinants, as well as the challenges associated with their clinical translation. This review aims to provide a framework for a deeper understanding of the complex biological basis of PTX resistance and to offer insights into the development of more precise therapeutic strategies for overcoming resistance.

## 1 Introduction

PTX is a naturally derived diterpenoid compound originally isolated from the bark of the Pacific yew tree. Since its clinical approval in the 1990s, PTX has become an important component of treatment regimens for a wide range of solid tumors (Zhai et al., 2023; Gaillard et al., 2025; Shi et al., 2021). Its mechanism of action has been well characterized: PTX primarily exerts its effects by binding reversibly to specific sites on β-tubulin, thereby promoting microtubule polymerization and inhibiting depolymerization. This disrupts microtubule dynamics, leading to cell cycle arrest at the G2/M phase and ultimately triggering apoptosis (Yu et al., 2025). Although PTX is widely used for the treatment of various solid tumors, both acquired and intrinsic resistance substantially limit its clinical efficacy, particularly in triple-negative breast cancer, in which the objective response rate is less than 50% (Zheng et al., 2024). Existing reviews have largely focused on individual mechanisms, such as drug efflux and microtubule alterations, whereas a systematic synthesis of the dynamic interactions among different resistance mechanisms and their regulatory network architecture remains lacking. Furthermore, increasing attention has recently been directed toward the roles of TME remodeling and metabolic reprogramming in PTX resistance; however, a unified framework for understanding these processes has yet to be established. Notably, despite growing insight into mechanisms involving drug efflux pumps, microtubule stability, and the TME, most resistance-reversal strategies have not produced satisfactory clinical outcomes. This limited success may be attributable to tumor heterogeneity, compensatory activation of multiple pathways, and the lack of precise biomarkers for patient stratification. Therefore, this review systematically integrates the core mechanisms of PTX resistance from multiple perspectives, with particular emphasis on the dynamic interactions among distinct resistance networks. It also summarizes current potential intervention strategies to overcome resistance, as well as the challenges associated with their clinical translation, with the aim of providing a theoretical foundation for the development of more precise therapeutic strategies.

## 2 Drug efflux pump mechanisms

Drug efflux pump activity is widely recognized as a key contributor to tumor cell resistance to PTX. Central to this process is the aberrant activation of members of the ATP-binding cassette (ABC) transporter superfamily, which markedly reduces the intracellular accumulation of PTX via ATP-dependent efflux (Choi, 2005). Within this superfamily, P-glycoprotein (P-gp/ABCB1), multidrug resistance-associated protein 1 (MRP1/ABCC1), and breast cancer resistance protein (BCRP/ABCG2) are the three major transporters most consistently implicated in PTX resistance. Through their distinct expression patterns and regulatory mechanisms, these transporters collectively constitute a critical efflux network underlying PTX resistance.

### 2.1 P-glycoprotein (P-gp/ABCB1)

P-gp is the most extensively studied mediator of multidrug resistance. Its aberrant overexpression has been closely associated with poor prognosis in multiple malignancies, including breast and ovarian cancers, and is inversely correlated with responsiveness to PTX therapy (Sauna and Ambudkar, 2000). Mechanistically, P-gp is often described as functioning as a “hydrophobic vacuum cleaner” or “hydrophobic vacuum pump.” After the lipophilic PTX molecule passively partitions into the plasma membrane, it binds to the substrate-binding region of membrane-embedded P-gp. This interaction subsequently triggers ATP hydrolysis at the nucleotide-binding domains, inducing conformational changes that drive substrate translocation from the inner leaflet or cytoplasmic side of the membrane to the extracellular space. Through this cyclical process, P-gp continuously lowers intracellular PTX accumulation, thereby attenuating its cytotoxic effects (Seelig and Li-Blatter, 2023).

P-gp overexpression is regarded as one of the most established molecular hallmarks of PTX resistance and is frequently observed in drug-resistant tumor cells and recurrent clinical specimens. A recent study Yang et al. (2024) reported that P-gp, together with BCRP and MRP1, is a key determinant of multidrug resistance, and that its overexpression is closely associated with reduced chemosensitivity and poor prognosis across multiple cancer types. *In vitro*, significantly elevated P-gp expression relative to parental sensitive cells has been detected in PTX-resistant cell lines, including high-grade serous carcinoma OVCAR8 cells (Nunes et al., 2021), breast cancer MCF-7/TAX cells (Chen et al., 2013), and lung cancer A549/TAX cells (Hu et al., 2026). Clinically, P-gp overexpression driven by ABCB1 translocations has been observed in approximately 20% of recurrent high-grade serous ovarian cancer samples, suggesting that ABCB1 rearrangement may represent an important molecular basis of acquired PTX resistance (Tighe et al., 2025). Taken together, evidence from both cellular models and clinical specimens supports a close association between aberrant P-gp activation and PTX resistance.

Beyond its upregulation, the ATP-dependent drug efflux activity of P-gp constitutes the core functional basis for its role in PTX resistance. As a known P-gp substrate, PTX can directly stimulate P-gp ATPase activity, thereby promoting energy-dependent efflux (Kimura et al., 2007). Treatment of resistant cells with P-gp inhibitors such as verapamil, elacridar, or tariquidar significantly restores sensitivity to PTX and markedly increases intracellular drug accumulation (Seelig and Li-Blatter, 2023). In the PTX-resistant ovarian cancer cell line NCI/ADR-RES, the P-gp inhibitor PGP-41 reduced the IC50 of PTX from 6.64 μM to 0.12 μM and increased intracellular PTX concentrations by more than tenfold (Sharma et al., 2023). Similarly, tyrosine kinase inhibitors such as gilteritinib (Zhang et al., 2024), lapatinib, and poziotinib (McCorkle et al., 2021) can also restore PTX cytotoxicity in resistant cells by directly inhibiting P-gp-mediated efflux. Collectively, these findings provide functional support for the contribution of P-gp-mediated efflux to PTX resistance.

A growing body of evidence indicates that aberrant P-gp activation is not an isolated event but is driven by the coordinated interplay of multilevel mechanisms, including transcriptional regulation, epigenetic reprogramming, and alterations in three-dimensional genome organization. P-gp expression is transcriptionally regulated by multiple upstream signaling pathways. On the one hand, in PTX-resistant lung cancer cells, activated Nrf2 directly binds to the ABCB1 promoter and upregulates its transcription. The natural flavonoid tangeretin effectively reverses this resistance by downregulating Nrf2, thereby suppressing ABCB1 transcription and P-gp-mediated PTX efflux, which restores chemosensitivity in lung cancer models (Xie et al., 2022). On the other hand, sustained activation of the STAT3 signaling pathway enhances MDR1/ABCB1 transcription, thereby promoting P-gp expression (Zhang et al., 2011). Activation of P-gp does not depend solely on local transcription factor binding but also involves changes in three-dimensional genome architecture. In PTX-sensitive cells, the ABCB1 locus resides within a transcriptionally repressed domain associated with the nuclear lamina and is therefore maintained in a silenced state. Upon acquisition of resistance, this genomic region dissociates from the nuclear lamina, and this spatial repositioning enables the transcriptional machinery to access the locus and initiate gene expression. This chromatin reorganization represents a critical upstream event in ABCB1 activation (Manjón et al., 2023). These findings suggest that ABCB1 activation depends not only on local transcriptional regulation but also on remodeling of the three-dimensional genome architecture.

### 2.2 Multidrug resistance-associated protein 1 (MRP1/ABCC1)

MRP1 is a member of the ABCC subfamily of ATP-binding cassette transporters and contributes to multidrug resistance by reducing the intracellular accumulation of a broad range of xenobiotics and anticancer agents. Unlike P-gp, MRP1 frequently transports organic anions and glutathione-, glucuronide-, or sulfate-conjugated compounds. The relationship between MRP1 and PTX, however, is more complex. PTX does not form a covalent PTX–GSH conjugate that is subsequently exported by MRP1. Instead, experimental evidence (Manciu et al., 2003) suggests that intracellular GSH may facilitate MRP1-associated drug transport through co-transport, allosteric modulation, or stabilization of a transport-competent conformation. Therefore, although MRP1 expression has been associated with PTX resistance in several experimental models, whether PTX is directly transported by MRP1 remains incompletely established and should be interpreted cautiously.

Multiple studies have demonstrated that PTX-resistant tumor cells frequently exhibit significant upregulation of MRP1, accompanied by enhanced drug efflux and decreased intracellular drug accumulation. A study established a multidrug-resistant osteosarcoma subline (SaOS-2_DoxR) through stepwise exposure to increasing concentrations of doxorubicin (Boichuk et al., 2023). This subline exhibited an approximately 30-fold increase in PTX resistance, which was mainly associated with overexpression of ABCB1 (P-gp) and ABCC1 (MRP1), leading to enhanced drug efflux. Further functional assays suggested that, although ABCB1 likely plays a dominant role in drug efflux in this model, MRP1 upregulation also contributes to the multidrug-resistant phenotype. Another study reported a predicted binding energy of −11.3 kcal/mol between PTX and an MRP1 structural model (Haque et al., 2022). However, molecular docking alone cannot establish that PTX is an authentic transported substrate, because ligand binding may occur at transport, regulatory, or allosteric sites without resulting in translocation. Accordingly, these computational findings should be regarded as hypothesis-generating and require validation using ATPase assays, vesicular transport assays, intracellular drug accumulation measurements, and genetic manipulation of MRP1. High-affinity binding does not establish that PTX is a transported substrate, as MRP1 also contains multiple allosteric sites that may bind compounds without affecting their translocation. Nevertheless, when considered alongside the functional efflux data, these structural models support the hypothesis that MRP1 upregulation may represent a candidate resistance-associated marker, although its predictive value requires validation in clinical cohorts, particularly in ovarian cancer contexts where the transporter is frequently overexpressed.

MRP1 expression is regulated by multiple upstream factors, including non-coding RNAs, sphingolipid metabolic enzymes, and the PI3K/Akt signaling pathway; dysregulation of these factors collectively contributes to MRP1-dependent PTX resistance. Previous studies have shown that certain long non-coding RNAs can competitively bind microRNAs through the competing endogenous RNA mechanism, thereby upregulating MRP1 expression (Mahabady et al., 2022). For example, LINC00518 has been reported to downregulate miR-199a, thereby relieving its inhibitory effect on MRP1 and ultimately promoting PTX resistance (Chang et al., 2018). In addition, LINC01118 has been shown to promote PTX resistance through regulation of the miR-134/ABCC1 axis, indicating that the lncRNA–miRNA–MRP1 network may represent an important post-transcriptional regulatory mechanism underlying PTX resistance (Shi and Wang, 2018). Recent study reported that long-term overexpression of ceramide synthase 4 (CerS4) in MCF-7 breast cancer cells upregulated multiple ABC transporter genes, including ABCC1 (MRP1), and promoted the acquisition of a drug-resistant phenotype (Kim et al., 2023). Conversely, CerS4 knockdown in resistant cells partially reversed ABCC1 overexpression and chemosensitivity loss, supporting the role of CerS4 as an upstream regulator of MRP1. Moreover, in colorectal cancer cells, shRNA-mediated Akt2 knockdown reduced the expression of MRP1 and MDR1 and enhanced cellular sensitivity to PTX, suggesting that aberrant activation of the PI3K/Akt pathway may also promote resistance through MRP1 upregulation (Ding et al., 2015). Collectively, these multilevel regulatory mechanisms indicate that MRP1-dependent resistance is not merely a consequence of enhanced drug efflux, but is also closely associated with adaptive remodeling of tumor cell redox balance, lipid metabolism, and survival signaling networks. Therefore, combinatorial interventions targeting the upstream regulatory network of MRP1 may provide promising therapeutic strategies for overcoming PTX resistance.

### 2.3 Breast cancer resistance protein (BCRP/ABCG2)

BCRP, encoded by ABCG2, is another member of the ABC transporter family. In contrast to P-gp, PTX is generally not regarded as a preferred or canonical BCRP substrate. Nevertheless, increased ABCG2 expression has been observed in several PTX-resistant or multidrug-resistant cell models, and genetic suppression of ABCG2 has restored PTX sensitivity in some experimental settings (Ramos et al., 2021; Türksoy Terzioğlu and Terzioğlu, 2026). These findings suggest that BCRP may contribute indirectly or conditionally to PTX resistance, depending on tumor lineage, co-administered drugs, transporter co-expression, and the broader multidrug-resistant phenotype. Compared with P-gp, BCRP exhibits distinct expression patterns and substrate selectivity across different tumor types. For example, both proteins are overexpressed in leukemia (Dei et al., 2019) while BCRP is more highly expressed in prostate cancer, whereas P-gp is more frequently associated with liver and kidney cancers (Ramos et al., 2021). The expression levels of these two proteins also differ among various subtypes of non-small cell lung cancer (Vesel et al., 2017). BCRP and P-gp exhibit substantial overlap in their substrate spectra. However, their substrate specificities are not completely identical. For example, BCRP transports methotrexate and mitoxantrone, which are not considered typical P-gp substrates (Noguchi et al., 2014). Furthermore, with respect to the physicochemical properties of transported molecules, P-gp predominantly transports neutral or positively charged hydrophobic compounds (Seelig, 2020). In contrast, BCRP displays a broader substrate spectrum and is capable of transporting a diverse range of organic anions, cations, and neutral compounds; moreover, its substrate recognition mechanisms appear to be more complex (Mao and Unadkat, 2015). These differences indicate that BCRP and P-gp should not be regarded as functionally interchangeable when designing anticancer strategies or predicting therapeutic responses; instead, their roles should be evaluated in the context of specific tumor types and drug properties.

Although BCRP is not the primary transporter involved in PTX efflux, its overexpression has been associated with PTX resistance under specific experimental conditions. Studies have shown that ABCG2 expression is significantly upregulated in PTX-resistant gastric cancer cells. These cells exhibit not only cross-resistance to cisplatin and 5-fluorouracil but also increased drug efflux capacity, invasiveness, spheroid-forming ability, and stem-like properties. Moreover, ABCG2 knockdown restores PTX sensitivity, suppresses drug-resistant and invasive phenotypes, and is associated with reduced ERK phosphorylation and decreased expression of downstream target genes (Türksoy Terzioğlu and Terzioğlu, 2026). Another study reported that A2780nirapR cells exhibited significant upregulation of BCRP and developed cross-resistance to PTX (Macdonald et al., 2026). Following stable shRNA-mediated knockdown of BCRP, the resistant cells regained sensitivity to PTX. Conversely, overexpression of ABCG2 in PTX-sensitive cells directly induced a resistant phenotype. In a stepwise PTX-selection model, ABCB1 and ABCG2 expression increased progressively with the level of resistance (Duz and Karatas, 2021). These findings suggest that elevated BCRP expression is closely associated with PTX resistance and that high ABCG2 expression may represent a candidate marker of PTX resistance in patients with laryngeal squamous cell carcinoma. In the established PTX-resistant breast cancer cell lines, MCF-7/PR and SKBR-3/PR, both BCRP mRNA and protein expression levels were significantly increased, accompanied by upregulation of other drug resistance-related genes, including MDR1 and MRP1. Transfection with a miR-21 inhibitor not only downregulated BCRP expression but also enhanced the sensitivity of resistant cells to PTX. This study suggests that BCRP expression is closely associated with PTX resistance and that miR-21 may contribute to the resistance mechanism by regulating drug resistance-related proteins such as BCRP (Zhao et al., 2015). Overall, available evidence supports an association between elevated BCRP expression and PTX resistance in selected experimental models. However, because PTX is not generally considered a canonical BCRP substrate and robust clinical evidence remains limited, BCRP should be regarded as a context-dependent contributor rather than a universal mediator of PTX resistance.

**FIGURE 1:**
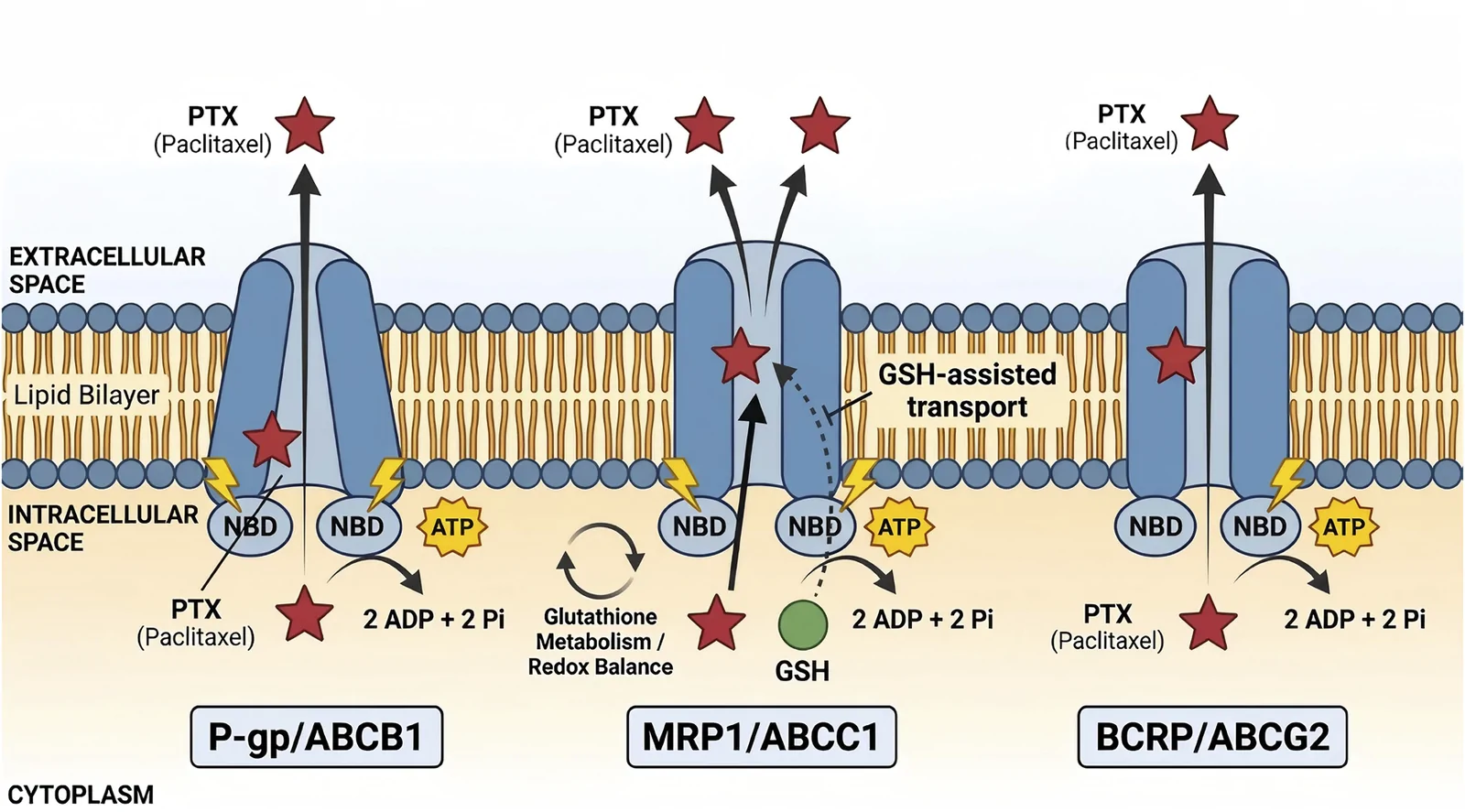
ATP-binding cassette transporter-associated mechanisms contributing to PTX resistance. P-gp directly mediates PTX efflux, whereas the contribution of MRP1 and BCRP is more context-dependent and may involve GSH-associated regulation, altered transporter expression, and cross-resistance phenotypes. Abbreviations: PTX, paclitaxel; P-gp, P-glycoprotein; MRP1, multidrug resistance-associated protein 1; BCRP, breast cancer resistance protein; GSH, reduced glutathione; NBD, nucleotide-binding domain.

Although P-gp, MRP1, and BCRP all belong to the ABC transporter family, they play distinct roles in mediating PTX resistance. P-gp primarily reduces the effective intracellular accumulation of PTX through ATP-dependent drug efflux, whereas MRP1 is more closely associated with GSH metabolism, cellular detoxification, and maintenance of redox homeostasis; in contrast, BCRP may contribute to acquired resistance and cross-resistance in selected cellular contexts. Together, these three proteins constitute a core efflux network underlying PTX resistance and, in conjunction with metabolic reprogramming, redox homeostasis, and cell survival signaling pathways, collectively sustain the resistant phenotype (Figure 1).

## 3 Tubulin- and microtubule-related mechanisms

**FIGURE 2:**
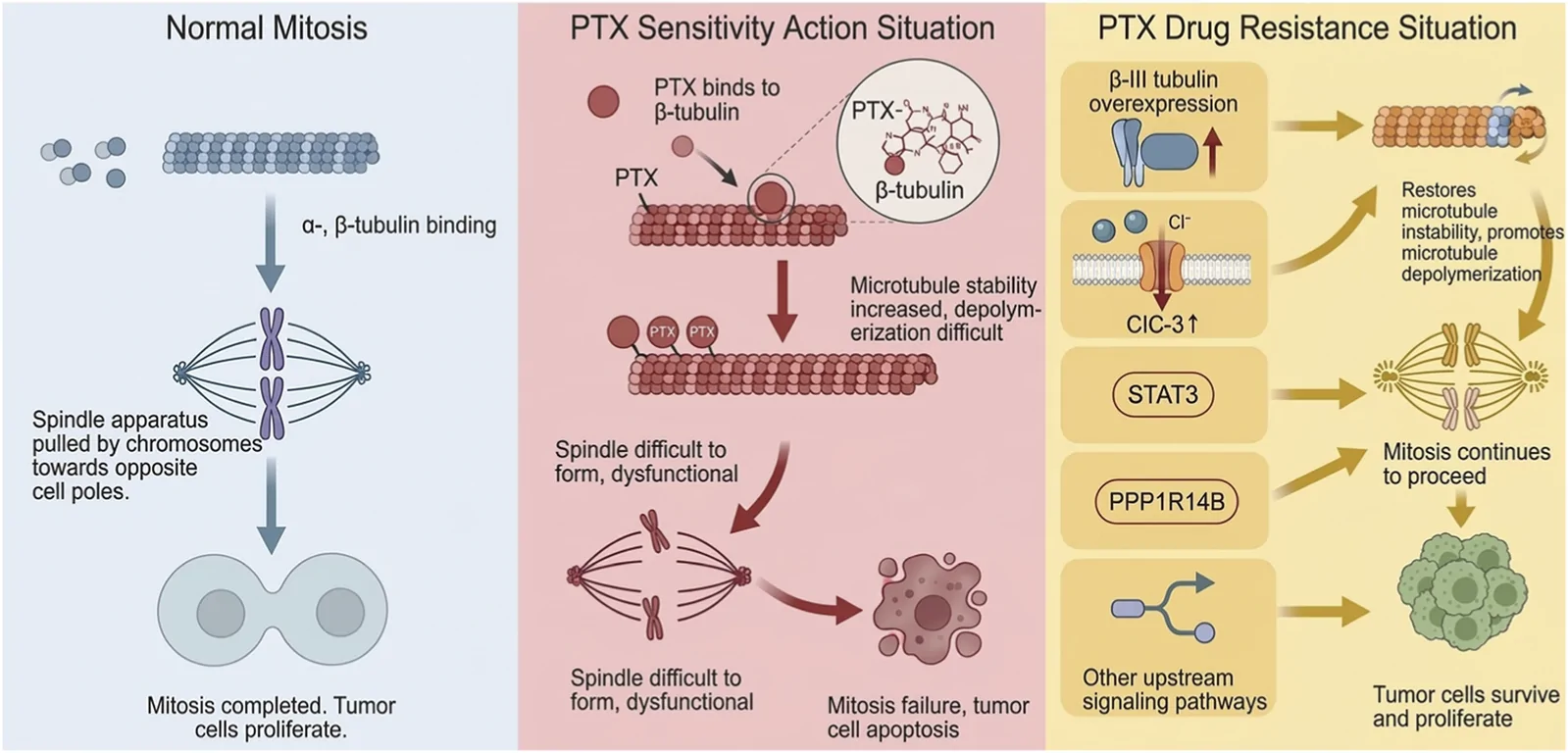
Tubulin- and microtubule-associated mechanisms underlying PTX resistance. Altered tubulin isoform expression, tubulin mutations, dysregulated microtubule-associated proteins, and upstream signaling networks collectively remodel microtubule dynamics and reduce PTX sensitivity. Abbreviations: PTX, paclitaxel.

Alterations in tubulin composition and microtubule dynamics represent another major mechanism of PTX resistance. These alterations include changes in β-tubulin isoform expression, tubulin mutations, dysregulation of microtubule-associated proteins, and adaptive remodeling of upstream signaling pathways. Together, these processes reduce the ability of PTX to stabilize microtubules and induce sustained mitotic arrest (Figure 2).

### 3.1 Alterations in tubulin isoform composition and microtubule dynamics

Microtubules are an essential component of the eukaryotic cytoskeleton, formed by the polymerization of α- and β-tubulins into heterodimers, resulting in a hollow tubular structure (Jordan and Wilson, 2004). Microtubules exhibit dynamic instability characterized by continuous polymerization and depolymerization, playing a critical role in maintaining cell morphology, intracellular transport, and mitosis. During prophase, microtubules polymerize to form the spindle apparatus and ensure the normal progression of cell division during chromosome separation (Sridhara and Shimamoto, 2024). Alterations in tubulin composition and kinetics are considered a major core mechanism driving PTX resistance, involving multiple levels. Overexpression of βIII-tubulin is the most common mechanism of PTX resistance; its accumulation enhances microtubule instability and reduces the binding stability of certain taxane drugs to microtubules, thereby weakening the drugs’ cytotoxic effects (Kanakkanthara and Miller, 2021). Other studies have indicated that tubulin mutation can directly block the binding of PTX to β-tubulin or reduce drug affinity by affecting the stability of α/β-heterodimers, thereby causing resistance (Zhou et al., 2024a). Another study in PTX-resistant ovarian cancer cells demonstrated that upregulation of the ClC-3 chloride channel protein allows it to directly bind to β-tubulin, disrupting microtubule polymerization homeostasis and reducing microtubule stability, thereby decreasing cellular sensitivity to PTX (Feng et al., 2021). Furthermore, the small-molecule tubulin-targeting PROTAC molecule W13 (Yang H. et al., 2024) effectively degrades α/β/β3-tubulin and overcomes PTX resistance in both *in vitro* and *in vivo* models, further highlighting the importance of maintaining microtubule homeostasis in drug resistance. In summary, alterations in tubulin isoform composition, genetic mutations, and imbalances in microtubule kinetic homeostasis can collectively reshape the response pattern of tumor cells to PTX and reduce drug sensitivity by enhancing microtubule adaptability. These studies further indicate that targeting the remodeling of microtubule homeostasis may represent an important intervention strategy for reversing PTX resistance.

### 3.2 Abnormal regulation of microtubule-associated proteins

Abnormal regulation of microtubule-associated proteins is also a key mechanism driving PTX resistance. Regarding microtubule motor proteins, a study found that PPP1R14B inhibits the microtubule-destabilizing function of stathmin 1 (STMN1) by maintaining its phosphorylated state, thereby altering microtubule homeostasis and promoting cell cycle progression (Liao et al., 2023), leading to PTX resistance in triple-negative breast cancer. Also focusing on STMN1 as a target molecule, another research found that STAT3 is persistently activated in drug-resistant ovarian cancer cells (Yang J. et al., 2024). Enhanced interaction between STAT3 and stathmin not only stabilizes microtubules but also promotes the transcription of pro-survival genes, thereby synergistically maintaining the drug-resistant phenotype. *In vitro* studies demonstrated that Tau protein shares β-tubulin binding sites with PTX; Tau can attenuate PTX-induced microtubule stabilization by reshaping microtubule assembly kinetics (Feizabadi and Castillon, 2022). Furthermore, a systematic analysis showed that α-tubulin dephosphorylation can enhance PTX sensitivity by inhibiting the activity of the microtubule depolymerase MCAK, suggesting that post-translational modifications of microtubules are also involved in the regulation of drug response (Lopes et al., 2023). In summary, microtubule-associated proteins can reshape the dynamic equilibrium of microtubules through multi-level mechanisms such as phosphorylation, protein interactions, and post-translational modifications, thereby enhancing the adaptive survival capacity of tumor cells under PTX stress and ultimately promoting the development of drug-resistant phenotypes.

### 3.3 Upstream adaptive signaling networks: a multilevel mechanistic landscape

In addition to alterations in tubulin itself and microtubule-associated proteins, accumulating evidence indicates that upstream adaptive signaling networks are also intimately involved in the development of PTX resistance. These mechanisms can be systematically categorized into five interconnected but distinguishable branches: non-coding RNA-mediated post-transcriptional regulation, kinase signaling-driven drug efflux, intercellular communication within the TME, cytoskeletal remodeling coupled with transcriptional reprogramming, and context-dependent innate immune modulation.

#### 3.3.1 Non-coding RNA-mediated regulation

Non-coding RNAs modulate PTX sensitivity by simultaneously targeting microtubule stability and drug penetration. A study in intrahepatic cholangiocarcinoma found that the circular RNA cPKM promotes PTX resistance by stabilizing stathmin 1 expression at the post-transcriptional level (Chen et al., 2023). Furthermore, cPKM promotes stromal fibrosis by stabilizing TGFB1 mRNA, thereby forming a physical barrier that impedes drug delivery, limits drug penetration, and exacerbates resistance. Beyond circRNAs, diverse non-coding RNA species—including lncRNAs and miRNAs—have been shown to regulate ABCB1, STMN1, apoptosis, and autophagy, broadening the regulatory repertoire (Guo and Cheng, 2022; Naseri et al., 2025; Tajik and Ghafouri-Fard, 2025; Liu et al., 2023). These findings indicate that non-coding RNAs can coordinate multiple resistance modules rather than regulating a single downstream target.

#### 3.3.2 Kinase signaling and transporter activation

Kinase signaling reprogramming intertwines with membrane transporter activity to regulate intracellular PTX accumulation. In pancreatic cancer, the transcription factor FoxM1 (specifically the FoxM1b and FoxM1c isoforms) directly binds to the promoter region of the PHB1 gene and upregulates its expression. PHB1 subsequently promotes the PHB1/C-RAF interaction and enhances the phosphorylation of ERK1/2 kinases, thereby activating the MAPK signaling pathway. Treatment with the FoxM1 inhibitor thiostrepton significantly reduces the expression of PHB1, p-ERK1/2, and ABCA2, increases the intracellular influx of paclitaxel, and attenuates FoxM1-mediated paclitaxel resistance both *in vitro* and *in vivo* (Huang et al., 2019). Although this example highlights ERK1/2, a broader network of kinase cascades—including PI3K/Akt, STAT3, and NF-κB—has been implicated in upregulating P-gp expression and sustaining cell survival, illustrating how kinase signaling converges on drug efflux and anti-apoptotic programs (Zhang et al., 2011; Abdin et al., 2021; Duarte and Vale, 2022).

#### 3.3.3 Extracellular vesicle-mediated intercellular communication

Small extracellular vesicles (sEVs) mediate intercellular communication by transferring diverse cargoes, including exosomal miRNAs, proteins, and lipids, thereby establishing a pro-resistance microenvironment through stromal reprogramming. Evidence from metastatic gastric cancer (Panzetta et al., 2025) revealed that patient-derived sEVs carry signaling molecules such as PDGFRβ, PPARγ, and various pro-angiogenic factors. Although these molecules do not directly interact with tubulin, they orchestrate profound phenotypic changes in recipient cells, including epithelial–mesenchymal transition (EMT) and extracellular matrix (ECM) remodeling. The resultant ECM stiffening and altered cell–matrix adhesion provide survival advantages that mitigate the apoptotic response normally triggered by microtubule disruption, creating a “protective niche” that indirectly sustains the resistant phenotype. Moreover, this vesicle-mediated cargo transfer can propagate resistance traits from drug-resistant to drug-sensitive cells, amplifying the resistant population within the tumor (Yan et al., 2024; Mao et al., 2024; Zhang et al., 2014).

#### 3.3.4 Cytoskeletal remodeling and epithelial–mesenchymal plasticity

Cytoskeletal remodeling, accompanied by EMT-related transcriptional reprogramming, reflects a global adaptive response to chronic PTX exposure. Researchers found that triple-negative breast cancer cells chronically exposed to PTX exhibited cytoskeletal remodeling characterized by γ-actin predominance, along with Twist1-mediated downregulation of E-cadherin and nuclear translocation of β-catenin (Dugina et al., 2024). This shift highlights a critical tubulin–actin cross-talk, in which the γ-actin-enriched cytoskeleton compensates for microtubule disruption and helps maintain cell integrity. The activation of this EMT transcriptional program reinforces cell plasticity and reduces reliance on stable microtubules for survival, effectively shifting the cellular equilibrium from a microtubule-centric vulnerability toward a more resilient, mesenchymal-like state that weakens the efficacy of PTX. Such plasticity is also associated with enhanced migration and invasion, further linking EMT-driven cytoskeletal remodeling to drug resistance.

#### 3.3.5 Context-dependent immune and stress signaling

Innate immune signaling, particularly the cGAS–STING pathway, exhibits a context-dependent dual role in PTX resistance that warrants careful distinction. Researchers demonstrated that the STING pathway is chronically activated in breast cancer cells that have acquired drug resistance (Hou et al., 2023). Far from triggering an antitumor immune response, this persistent activation actually sustains the survival of cells exhibiting chromosomal instability through adaptive low-grade inflammation, thereby actively promoting the maintenance of drug resistance. In stark contrast, acute STING activation enhances antitumor immunity, and the novel microtubule destabilizer S-72 exploits this mechanism by inhibiting STING activity to induce lethal chromosomal instability. Therefore, whether STING signaling promotes or suppresses drug resistance depends largely on the duration of activation, tumor type, and the prevailing state of the immune microenvironment, rather than reflecting a static, unidirectional regulatory mechanism (Gong et al., 2026; Khorramdelazad et al., 2026; Majaz et al., 2026). This pronounced context dependency cautions against generalizing STING function across different cancers and treatment settings.

Collectively, these upstream networks do not function as isolated linear pathways. Instead, they converge on drug efflux, microtubule remodeling, cell-death avoidance, and tumor–stroma communication. Their relative contribution is highly dependent on tumor lineage and molecular context. A comprehensive dissection of these distinct branches is essential for designing rational combination strategies that target specific resistance nodes in individual tumor contexts.

## 4 Other key mechanisms of drug resistance: adaptive survival and maintenance of resistance

The preceding sections have systematically described the central roles of drug efflux pumps and tubulin in PTX resistance; however, these mechanisms do not operate in isolation, but are instead intricately interconnected through complex signaling networks. In addition to these two core mechanisms, tumor cells evade PTX-induced cell death by activating downstream or bypass survival pathways, primarily involving dysregulation of apoptosis and autophagy, remodeling of the TME, metabolic reprogramming, and epigenetic as well as transcriptional alterations. Together, these multilevel mechanisms constitute a networked foundation for the development and long-term maintenance of PTX resistance (Table 1).

**TABLE 1:** Context-dependent mechanisms of paclitaxel resistance across major tumor types.

Tumor type	Experimental model(s)	Resistance mechanism(s)	Key molecule/pathway(s)	Evidence level	Reversal strategy(s)
Ovarian cancer	NCI/ADR-RES, SKOV3-TR, A2780/T, patient specimens	P-gp-mediated efflux; MRP1-associated transport; protective autophagy via PNPO/LAMP2; ECM remodeling (COL5A1); NF-κB activation	P-gp, MRP1, PNPO, COL5A1, NFKBIE	Cell-line; patient-tissue association (COL5A1)	P-gp inhibitor (PGP-41); PNPO knockdown; COL5A1 knockdown; TGT combination
Breast cancer	MCF-7/TXL, MDA-MB-231, MCF-7/PR, SKBR-3/PR	P-gp/BCRP efflux; FOXO1-driven protective autophagy; CAF-induced integrin β1 signaling; actin cytoskeleton remodeling (γ-actin predominance)	P-gp, BCRP, FOXO1, integrin β1, γ-actin	Cell-line; animal (FOXO1 xenograft)	P-gp inhibitors; miR-21 inhibitor; autophagy inhibitor (3-MA); HA-oligosaccharide nanoparticles
Non-small cell lung cancer	A549/T	P-gp efflux; Nrf2-mediated ABCB1 transcriptional upregulation	P-gp, Nrf2	Cell-line	Nrf2 inhibitor (tangeretin)
Gastric cancer	PTX-resistant gastric cancer cells; CAF co-culture models	BCRP upregulation; CAF-derived miR-522 suppressing ferroptosis (ALOX15); sEV-mediated EMT and ECM remodeling	BCRP, miR-522, ALOX15	Cell-line	BCRP knockdown; miR-522 inhibition
Pancreatic cancer	PDAC cell lines; xenograft models	HIF-1α/AVL9/NF-κB axis; circERC1-mediated ECM remodeling; Hedgehog pathway-driven stromal desmoplasia	AVL9, circERC1, Hedgehog	Cell-line; animal (Hedgehog inhibitor)	AVL9 targeting; circERC1 inhibition; cyclopamine/PTX co-delivery nanocarrier
Nasopharyngeal carcinoma	NPC cell lines	CENPN-mediated suppression of autophagic cell death via VAMP8 downregulation	CENPN, VAMP8	Cell-line	CENPN knockdown
Osteosarcoma	SaOS-2_DoxR (doxorubicin-selected)	Co-overexpression of P-gp and MRP1 leading to multidrug resistance	P-gp, MRP1	Cell-line	Not specifically tested in this model; general inhibitors apply

Evidence levels are defined as follows: Cell-line evidence – findings from established cancer cell lines *in vitro*; Animal evidence – results from *in vivo* xenograft or orthotopic models; Patient-tissue association – correlative analyses in patient specimens (e.g., immunohistochemistry, RNA sequencing); Clinical evidence – data from clinical trials or patient cohorts (none identified in the reviewed studies).

### 4.1 Apoptosis escape and dysregulation of autophagy

Evasion of apoptosis and dysregulation of autophagic homeostasis represent two intertwined but conceptually distinct adaptive hallmarks that enable tumor cells to withstand PTX-induced cytotoxicity. However, the precise functional outcome of autophagy—whether it serves as a survival shield or a death executor—is highly context-dependent, varying not only across cancer types but also according to the specific molecular lesions within each tumor. To dissect this complexity, the underlying regulatory mechanisms can be systematically categorized into three operational layers: transcriptional activation of autophagosome formation, enhancement of lysosomal degradation flux, and modulation of autophagosome–lysosome fusion. Importantly, these layers do not uniformly promote resistance; rather, their net contribution to PTX sensitivity depends on whether the resultant autophagic activity alleviates or exacerbates cellular stress.

Transcriptional upregulation of autophagic initiation constitutes a classic pro-survival adaptive response to PTX. In triple-negative breast cancer MDA-MB-231 cells, PTX simultaneously triggers both apoptotic signals and autophagic flux. Mechanistically, the transcription factor FOXO1 plays a pivotal role in this process by directly binding to the promoter regions of key autophagy-related genes, including ATG5, BECN1 (encoding Beclin-1), and VPS34. The transcriptional activation of these genes promotes the assembly of the Class III PI3K complex, which nucleates autophagosome formation. This FOXO1-driven autophagic response is protective rather than destructive; by clearing damaged organelles and recycling metabolic precursors, it raises the cellular stress threshold and delays the onset of mitochondrial outer membrane permeabilization. Consequently, pharmacological inhibition of autophagy using 3-methyladenine (3-MA) or genetic knockdown of FOXO1 significantly enhances PTX-induced apoptosis, as evidenced by increased caspase-3 cleavage and PARP degradation (Xu et al., 2022). *In vivo* xenograft models further corroborated this synergy, demonstrating that combined 3-MA and PTX treatment effectively suppresses tumor growth while amplifying apoptotic signatures within tumor tissues (Wang, J. et al., 2025a), which illustrates that, in certain contexts, autophagy acts as a rheostat that dampens the apoptotic response to microtubule disruption.

A distinct layer of autophagic regulation operates at the lysosomal degradation stage, where sustained autophagic flux fuels resistance by ensuring efficient clearance of autophagic cargo. In PTX-resistant ovarian cancer cells, enhanced autophagic flux is not primarily driven by initiation signals but rather by increased lysosomal proteolytic capacity. Pyridoxamine 5′-phosphate oxidase (PNPO), which is significantly overexpressed in these resistant cells, potentiates lysosomal function through the TFEB/LAMP2 transcriptional axis. Specifically, PNPO upregulates LAMP2 expression via TFEB-mediated lysosomal biogenesis, thereby accelerating the fusion of autophagosomes with lysosomes and the subsequent degradation of sequestered materials. This heightened flux provides a robust survival advantage by maintaining metabolic homeostasis and preventing the accumulation of toxic protein aggregates under PTX-induced proteotoxic stress. Disruption of this terminal degradation step—either by PNPO knockdown or by chloroquine-mediated blockade of autophagosome–lysosome fusion—effectively halts autophagic flux and restores PTX sensitivity. These findings uncover a novel resistance axis in which the PNPO–LAMP2 pathway sustains resistance specifically by augmenting the degradative capacity of the autophagy machinery (Li et al., 2024).

In certain cancers, autophagy itself can be a death-promoting mechanism, and its suppression paradoxically drives PTX resistance. This is exemplified in nasopharyngeal carcinoma, where PTX treatment upregulates VAMP8, a critical SNARE protein that mediates autophagosome–lysosome fusion. Enhanced VAMP8-driven fusion leads to an overwhelming autophagic flux that exceeds the cellular buffering capacity, ultimately triggering autophagic-dependent cell death — a non-apoptotic form of regulated cell death. In this context, the resistance phenotype emerges from the aberrant overexpression of centromeric protein N (CENPN), which represses the CREB/VAMP8 signaling axis. By inhibiting VAMP8 transcription, CENPN effectively suppresses autophagosome maturation and fusion, thereby blocking ADCD and conferring resistance to PTX. Consequently, genetic silencing of CENPN restores VAMP8 expression, reinstates autophagic activity, and successfully resensitizes resistant NPC cells to PTX (Wang, B. R. et al., 2024). This mechanistic branch stands in stark contrast to the TNBC and ovarian cancer models, as it frames autophagy as a vulnerability to be exploited rather than a survival pathway to be inhibited.

**FIGURE 3:**
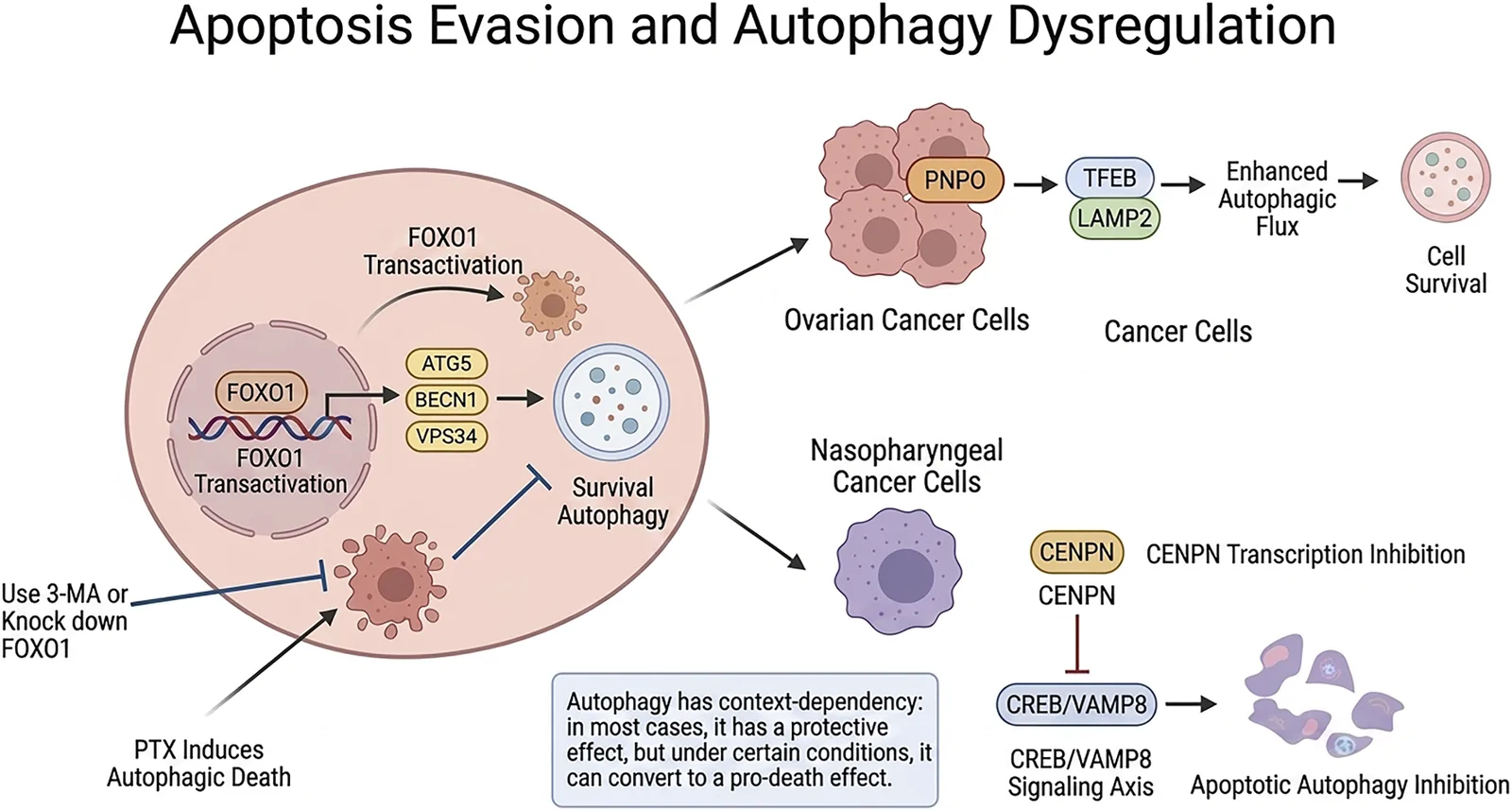
Context-dependent interplay between apoptosis evasion and autophagic regulation in PTX resistance. Autophagy may either protect tumor cells from PTX-induced stress or promote cell death, depending on tumor lineage and the dominant molecular regulatory network. Abbreviations: PTX, paclitaxel.

Notably, the autophagic state is not static; rather, tumor cells exhibit remarkable plasticity in transitioning between protective and lethal autophagy in response to varying durations of PTX exposure or concomitant stresses (Figure 3). This plasticity implies that sweeping strategies aimed at globally augmenting or suppressing autophagy are unlikely to yield consistent therapeutic benefits. Instead, a more precise therapeutic approach would be to identify and selectively target the dominant lineage- or context-specific regulatory nodes. Such interventions may shift autophagy from a cytoprotective to a cytotoxic state or release apoptotic pathways from inhibitory control, thereby improving the durability of PTX-resistance reversal.

### 4.2 Remodeling of the TME

**FIGURE 4:**
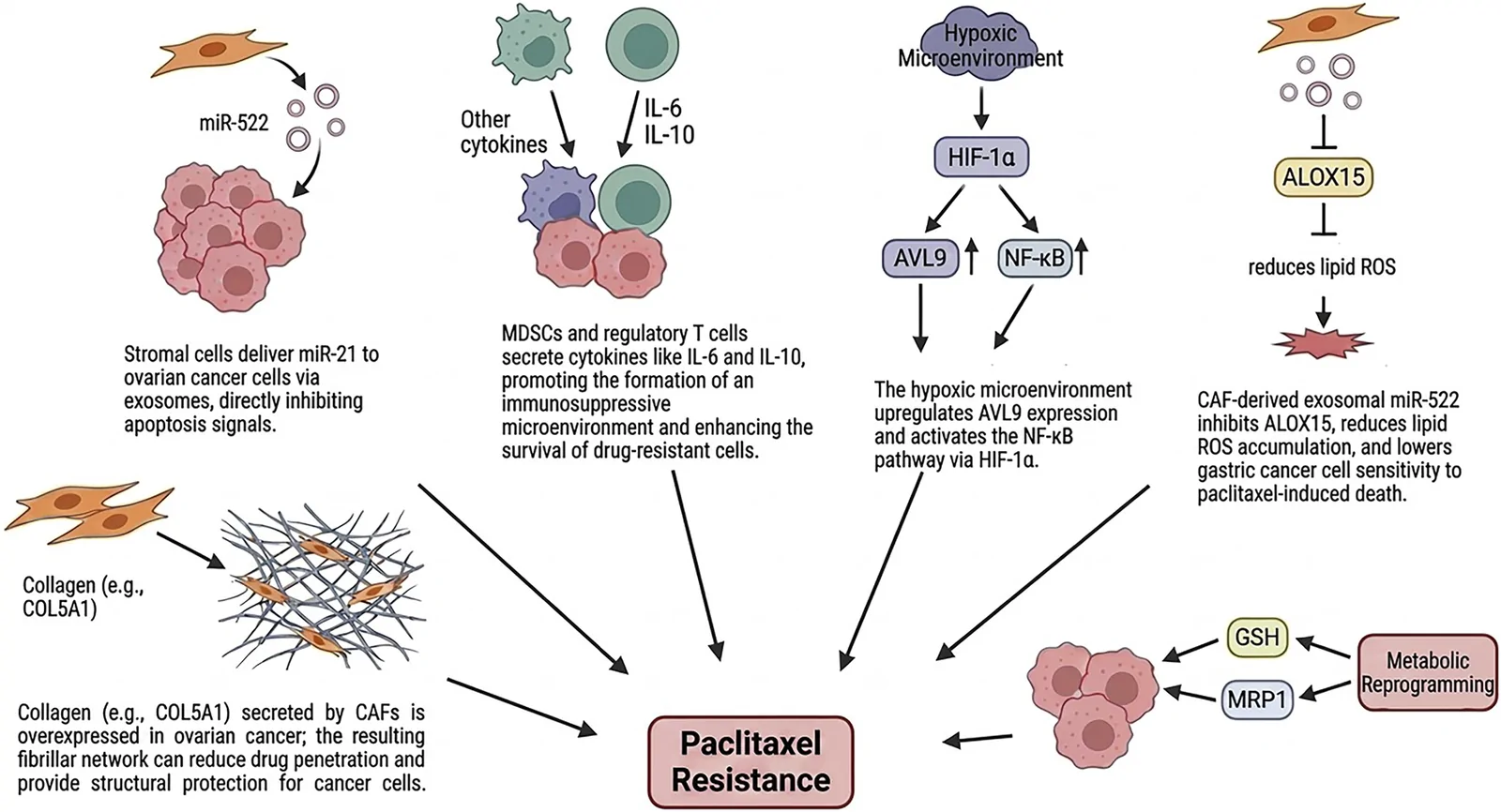
TME and metabolic mechanisms of PTX resistance. Abbreviations: PTX, paclitaxel; MRP1, multidrug resistance-associated protein 1; GSH, reduced glutathione.

Remodeling of the TME is a major extrinsic driver of PTX resistance, with aberrant immune cell polarization and the establishment of an immunosuppressive state playing particularly critical roles. Studies have shown that stromal cells within the TME can deliver miR-21 to ovarian cancer cells via exosomes, thereby suppressing apoptotic signaling and promoting chemoresistance (Au Yeung et al., 2016). In addition, myeloid-derived suppressor cells can promote chemoresistance by suppressing antitumor immune responses and releasing inflammatory or immunoregulatory mediators (Lasser et al., 2024). Regulatory T cells may further contribute to an immunosuppressive microenvironment that permits the persistence of resistant tumor cells (Jiang et al., 2026). However, direct evidence linking these immune populations specifically to PTX resistance remains limited and appears to be tumor-context dependent. Beyond immunosuppression, metabolic reprogramming represents another crucial link between TME adaptation and drug resistance. A hypoxic microenvironment can upregulate AVL9 expression via HIF-1α and activate the NF-κB pathway, thereby promoting pancreatic cancer resistance to combined PTX/gemcitabine therapy (Ding, J. et al., 2025). In addition, the one-carbon metabolism-related gene SFXN3 is highly expressed in head and neck squamous cell carcinoma and is associated not only with tumor progression, but also potentially with PTX resistance and the establishment of an immunosuppressive microenvironment (Chen, K. et al., 2022). Furthermore, metabolism-related factors such as lactate and lipid peroxides can influence tumor cell sensitivity to PTX through regulation of ferroptosis. For example, cancer-associated fibroblasts (CAFs) suppress ALOX15 via exosomal miR-522, thereby reducing lipid ROS accumulation and decreasing the sensitivity of gastric cancer cells to PTX-induced cell death (Zhang, H. et al., 2020). Moreover, remodeling of the ECM and fibrotic networks within the TME can create physical barriers that limit drug penetration and provide structural protection for resistant cells. For instance, in ovarian cancer, COL5A1 overexpression is directly associated with PTX resistance, and knockdown of COL5A1 restores PTX sensitivity (Zhang, J. et al., 2021). In breast cancer, however, collagen derived from CAFs can induce PTX resistance via activation of the integrin β1/PI3K/AKT signaling pathway (Li et al., 2019). In addition, the remodeling of the ECM and fibrotic networks within the TME can form physical barriers that limit drug penetration and provide structural protection for drug-resistant cells. To overcome this barrier, nanoparticle-based drug delivery systems that target and modulate ECM components have demonstrated the potential to partially reverse PTX resistance in experimental models. Specific mechanisms include degrading the physical ECM barrier, modulating cancer-associated fibroblast (CAF)-driven ECM remodeling, and reshaping the ECM structure. First, degrading key barrier components within the ECM can effectively enhance drug penetration. Studies have demonstrated that the hyaluronic acid (HA) “coating” enriched on the surface of breast cancer cells acts as a physical barrier, hindering drug entry into tumor tissue (Yang et al., 2013). The construction of self-assembled nanoparticles loaded with HA oligosaccharides (oHA) can effectively degrade the HA matrix associated with tumor cells, rendering previously drug-resistant breast cancer cells significantly more sensitive to PTX. In a mouse tumor model, systemic administration of oHA nanoparticles not only reduced HA accumulation in the ECM surrounding xenografts but also significantly enhanced the chemotherapeutic activity of PTX and inhibited tumor growth, providing direct evidence for overcoming HA-related chemoresistance. Second, targeting CAF-mediated ECM remodeling can indirectly improve drug distribution and attenuate the protective effect of the matrix on tumor cells. Based on the key role of the PAK1 signaling pathway in CAF differentiation, migration, and contraction, one study employed electrospun polycaprolactone nanofibers to co-deliver the PAK1 inhibitor (FRAX597) and PTX (A et al., 2024). In a 3D heterogeneous tumor spheroid model rich in matrix, PTX-containing nanofibers alone exhibited limited efficacy due to the matrix barrier, whereas nanofibers co-delivering PTX and FRAX597 reduced tumor spheroid growth and activity by more than 90%; this effect was closely associated with decreased expression levels of collagen I and α-SMA within the spheroids. This finding suggests that remodeling the ECM by inhibiting CAF activity represents an effective strategy for enhancing PTX efficacy. Furthermore, remodeling the physical structure and mechanical properties of the ECM also contributes to overcoming drug resistance. The researchers developed a polymer micelle formulation that simultaneously delivers the Hedgehog signaling pathway inhibitor cyclopamine (CPA) and PTX (Zhao, J. et al., 2018). In a pancreatic ductal adenocarcinoma (PDAC) model, CPA effectively modulated the tumor stroma by depleting matrix-producing CAFs, as evidenced by increased microvascular density, alleviated hypoxia, and reduced stromal stiffness, while preserving the tumor-restraining function of the ECM. This stromal remodeling approach improved drug distribution within the tumor, enabling the M-CPA/PTX combination therapy to significantly inhibit tumor growth and prolong the survival of tumor-bearing animals. Overall, the TME contributes to the development of drug resistance through immune suppression, metabolic reprogramming, and ferroptosis regulation, while maintaining the PTX-resistant phenotype through the construction of a protective ecological network via ECM remodeling. Notably, metabolic reprogramming may also intersect with drug efflux mechanisms by modulating GSH metabolism and MRP1 function, thereby further enhancing PTX resistance (Figure 4).

### 4.3 Epigenetic regulation and gene expression

**FIGURE 5:**
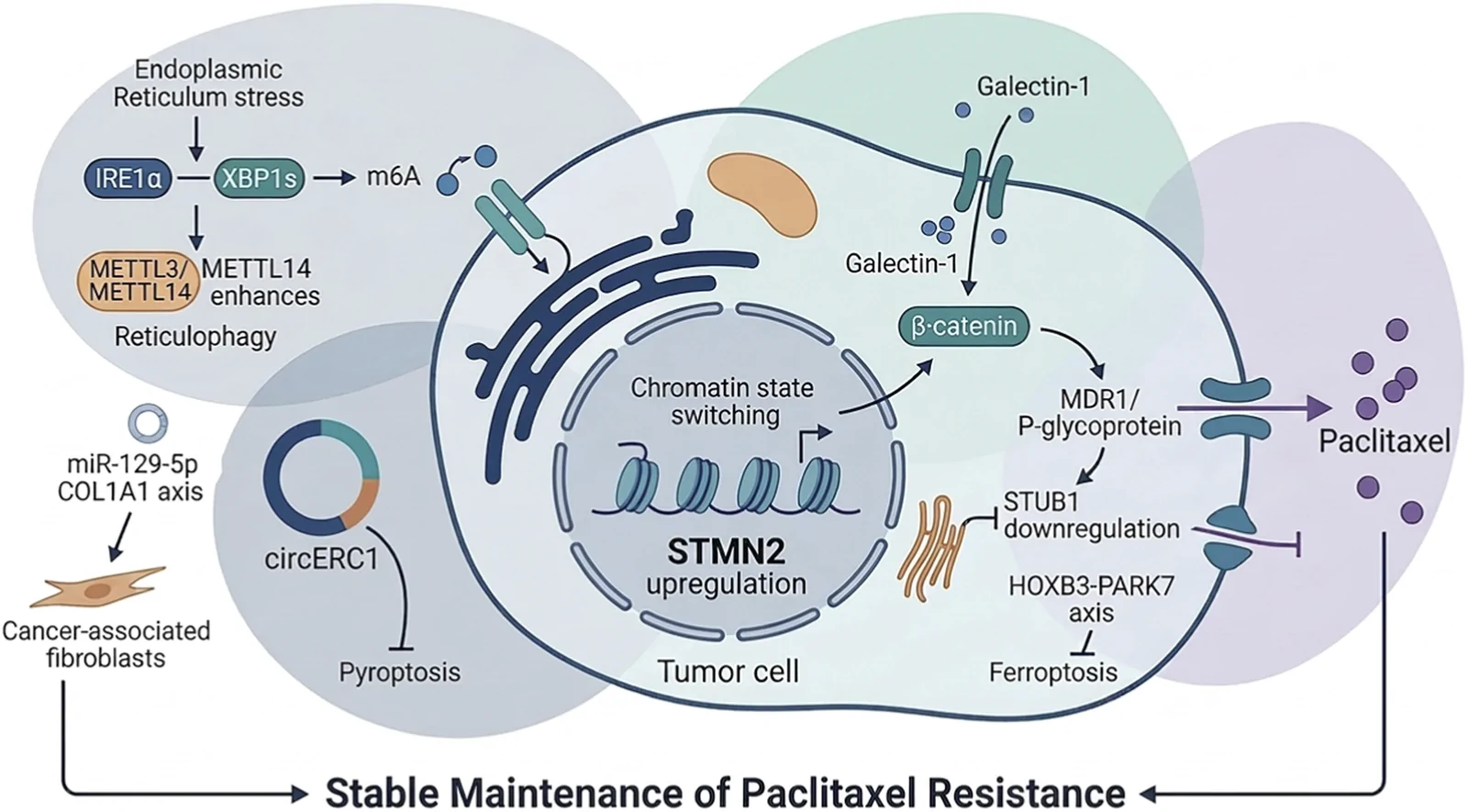
Epigenetic and transcriptional regulatory mechanisms of PTX resistance.

A growing body of evidence indicates that PTX resistance involves not only genetic alterations, but is also closely associated with epigenetic reprogramming. The underlying mechanisms operate at multiple levels, including alterations in chromatin states, RNA modifications, non-coding RNA-mediated regulation, and post-translational protein modifications. In lung adenocarcinoma cells, PRMT5 inhibitors induce stable resistance through drug-induced transcriptional state switching. This epigenetic transition is accompanied by upregulation of the microtubule-regulating protein stathmin 2 (STMN2). STMN2 is not only essential for maintenance of the resistant state, but also confers acquired sensitivity to PTX, suggesting that epigenetic state switching can alter tumor cell responsiveness to PTX by reshaping microtubule dynamics. Clinical data further suggest that high STMN2 expression is significantly associated with complete remission in patients treated with taxanes (Mueller et al., 2021). At the level of non-coding RNA regulation, researchers found that PTX treatment upregulates the circular RNA circERC1 in pancreatic cancer (Zhang, J. et al., 2025). CircERC1 suppresses pyroptosis-related pathways by regulating the stability of RNA-binding proteins. Concurrently, tumor cells can deliver circERC1 to CAFs via exosomes and remodel the ECM through the miR-129-5p/COL1A1 axis, thereby promoting PTX resistance through both suppression of immunogenic cell death and reinforcement of the ECM barrier. At the level of post-translational protein regulation, studies have shown that downregulation of the E3 ubiquitin ligase STUB1 in resistant ovarian cancer leads to aberrant accumulation of HOXB3 and promotes transcriptional activation of PARK7, which in turn induces PTX resistance through inhibition of ferroptosis. These findings suggest that ferroptosis evasion mediated by the STUB1–HOXB3–PARK7 axis may represent an important mechanism for maintenance of resistance (Zhao, L. et al., 2024). In esophageal squamous cell carcinoma, Galectin-1 is highly expressed in resistant cells and secreted into the extracellular space. By promoting nuclear translocation of β-catenin, Galectin-1 activates MDR1 transcription downstream of the Wnt/β-catenin pathway, thereby enhancing P-gp-mediated PTX efflux. Conversely, blockade of Galectin-1 partially reverses chemoresistance, suggesting that it may serve as a potential therapeutic target (Zhou et al., 2024b). At the level of RNA modification, PTX induces endoplasmic reticulum (ER) stress in breast cancer cells and, through the IRE1α–XBP1s signaling axis, increases m6A RNA methylation, thereby enhancing the transcript stability of ER-phagy-related molecules. By accelerating clearance of damaged ER, this mechanism maintains cellular homeostasis and enhances the adaptive survival capacity of cancer cells under PTX stress. Conversely, pharmacological inhibition or genetic ablation of METTL3/METTL14 enhances cellular sensitivity to PTX by blocking this adaptive autophagy pathway (Wang J. et al., 2024). Overall, epigenetic reprogramming does not promote PTX resistance through a single pathway; rather, it dynamically reshapes tumor cell survival states, metabolic adaptation, and drug-response networks through multilayered mechanisms—including RNA modifications, non-coding RNA regulation, and post-translational protein modifications—that collectively stabilize the resistant phenotype (Figure 5).

## 5 Therapeutic strategies and translational challenges for overcoming PTX resistance

### 5.1 Multi-mechanism interventions based on drug delivery systems

Nanomedicine-based delivery systems represent a promising strategy for overcoming PTX resistance by enhancing intratumoral drug accumulation, enabling co-delivery of chemotherapeutics and gene therapeutics, and simultaneously targeting resistance mechanisms such as drug efflux, autophagy, and microtubule homeostasis. One study developed a nanoassembly in which PTX was conjugated to polyethyleneimine, while small interfering RNA (siRNA) targeting Mdr1 was simultaneously encapsulated. This system silences Mdr1 expression through RNA interference, thereby reducing P-gp-mediated drug efflux. In parallel, the polyethyleneimine component alkalizes lysosomes, blocks autophagosome–lysosome fusion, and inhibits protective autophagy, thereby interfering with non-efflux-mediated resistance mechanisms. By simultaneously targeting efflux pumps and protective autophagy, this strategy significantly inhibited growth of drug-resistant lung cancer in both *in vitro* and *in vivo* models, suggesting that multi-mechanism co-delivery systems hold substantial promise for restoring PTX sensitivity in resistant tumors (Wang C. et al., 2022). Another approach employs multifunctional mesoporous polydopamine nanoparticles for co-delivery of PTX and siRNA targeting STMN1. Because elevated STMN1 expression promotes microtubule depolymerization, it antagonizes PTX-induced microtubule stabilization and thereby contributes to chemoresistance. By silencing STMN1, this nanosystem restores microtubule stability, enhances PTX sensitivity, and synergistically induces ovarian cancer cell death (Qian et al., 2025). To address blood–brain barrier constraints and drug resistance in glioma, another study developed PTX-loaded mesoporous selenium nanoparticles fused with C6 cell membranes and modified with the Angiopep-2 (Ang-2) targeting peptide. This system elevates intracellular reactive oxygen species, thereby inducing oxidative stress and mitochondrial dysfunction. It also promotes a shift from cytoprotective autophagy toward pro-death autophagic signaling. Pharmacological inhibition of autophagy attenuates this effect, suggesting that the nanosystem promotes cell death, at least in part, through modulation of autophagic flux. This strategy highlights the therapeutic potential of synergistically integrating PTX-based chemotherapy with oxidative stress induction and autophagy–apoptosis regulation (Shi H. et al., 2025). Overall, the major advantage of nanodelivery systems lies not only in enhancing local PTX accumulation, but also in their capacity to integrate multiple anti-resistance strategies simultaneously, including efflux pump inhibition, microtubule homeostasis modulation, autophagy reprogramming, and biological barrier penetration.

### 5.2 Combination therapy and network-based interventions for drug resistance

Combination therapy has emerged as a key strategy for improving responses to PTX by simultaneously targeting multiple resistance-associated pathways, and numerous preclinical studies have explored the combination of PTX with agents acting through distinct mechanisms. In ovarian cancer models, the combination of asparagus extract with low-dose PTX exerts synergistic inhibitory effects in both PTX-sensitive and PTX-resistant cells, and this synergy may be associated with enhanced DNA damage signaling and suppression of microtubule dynamics. Notably, this regimen elicits a distinct molecular response pattern in resistant cells compared with sensitive cells: asparagus extract inhibits PTX-induced MDR1 overexpression and suppresses activation of the AKT/mTOR/S6 pathway in resistant cells. These findings suggest that this combination is not merely additive, but may restore sensitivity to low-dose PTX by reshaping the resistance-associated molecular phenotype (Zhang, X. et al., 2023). In endometrial cancer, another study found that the RNA-binding protein IGF2BP3 is aberrantly overexpressed in PTX-resistant cells. Mechanistically, IGF2BP3 promotes c-Myc accumulation by inhibiting SPOP-mediated ubiquitination and degradation of c-Myc, whereas c-Myc in turn enhances IGF2BP3 transcription, thereby establishing an IGF2BP3/c-Myc positive feedback loop that sustains the resistant state. Based on this mechanism, researchers combined the c-Myc inhibitor 10058-F4 with PTX. Inhibition of c-Myc restored PTX sensitivity in resistant cells in both *in vitro* and *in vivo* models. This study suggests that disrupting positive feedback circuits that maintain resistance may represent an important combination strategy for improving PTX efficacy (Ge et al., 2025). In addition, combinations of PTX with traditional medicines or natural product-derived inhibitors have also shown potential for overcoming resistance. For example, Tongguanteng Injection (TGT), an aqueous extract derived from the traditional Chinese medicinal plant *Marsdenia tenacissima*, has been reported to reverse PTX resistance in ovarian cancer models. The combination of TGT and PTX exhibited synergistic effects in both A2780 cells and the resistant A2780/T subline, reducing the IC50 of PTX by 22.5-fold and 5.44-fold, respectively. The reversal of resistance may be associated with upregulation of TAB1 and activation of the TAB1/TAK1/p38 MAPK signaling pathway. Furthermore, TGT enhances the antitumor activity of PTX in nude mouse xenograft models, suggesting that integration of traditional herbal preparations with modern chemotherapeutic agents may improve PTX responsiveness through regulation of specific signaling pathways, thereby providing a potential strategy for overcoming resistance (Kong et al., 2023). Overall, the major advantage of combination therapy lies in its ability to simultaneously target efflux mechanisms, survival signaling, and phenotypic reprogramming in resistant cells; however, its efficacy remains highly dependent on tumor context and the dominant resistance mechanisms.

### 5.3 Novel molecular targets and pathway interventions

In recent years, targeting key molecules and signaling pathways involved in drug resistance has emerged as an important strategy for overcoming PTX resistance. Nuclear factor κB inhibitor epsilon (NFKBIE) is an endogenous negative regulator of the NF-κB signaling pathway. In PTX-resistant ovarian cancer cell lines, downregulation of NFKBIE leads to sustained NF-κB activation. Activated NF-κB promotes transcription of pro-survival genes such as BCL2 and XIAP, suppresses the mitochondrial apoptotic pathway, and induces MDR1 expression, thereby enhancing PTX efflux (Li Y. et al., 2024). In the PTX-resistant ovarian cancer cell line SKOV3-TR, NFKBIE overexpression inhibits NF-κB nuclear translocation and downregulates MDR1 protein expression, thereby restoring PTX sensitivity (Ding Q. et al., 2022). These findings suggest that restoring endogenous negative regulatory mechanisms may represent a viable strategy for overcoming resistance. In addition, another study identified miR-200b-3p as another potential therapeutic target (Wang J. et al., 2025b). This microRNA downregulates the drug-metabolizing enzyme CYP1B1 at the post-transcriptional level, thereby increasing PTX sensitivity in hepatocellular carcinoma. Further *in vivo* experiments demonstrated that miR-200b-3p agonists reduced CYP1B1 protein levels in tumor tissues and enhanced both antitumor efficacy and microtubule polymerization when combined with PTX, suggesting that the miR-200b-3p/CYP1B1 axis may sensitize tumors by reinforcing PTX-induced microtubule stabilization. Overall, the therapeutic value of emerging molecular targets lies not only in regulating individual resistance-associated genes, but also in disrupting the signaling networks that maintain the resistant phenotype through intervention at key nodes such as NF-κB, MDR1, CYP1B1, and microtubule regulatory pathways. In the future, molecularly stratified, multi-target therapeutic strategies may prove more effective for improving responses in PTX-resistant tumors.

## 6 Conclusions and outlook

PTX resistance is a complex and dynamic process driven by multiple mechanisms, including enhanced drug efflux, microtubule remodeling, evasion of apoptosis, dysregulation of autophagy homeostasis, adaptation of the TME, and epigenetic reprogramming. Among these mechanisms, ABC transporter-mediated drug efflux and microtubule abnormalities remain the two canonical drivers of resistance. Intervention strategies based on nanodelivery systems, combination therapy, and emerging molecular targets provide new opportunities for multi-level modulation of the resistance network. Future research on PTX resistance should move beyond inhibition of individual resistance factors and instead focus on the dynamic remodeling of the resistance network, with particular attention to the coordinated interplay among drug efflux, microtubule homeostasis, metabolic adaptation, epigenetic plasticity, and the TME. Emerging technologies such as single-cell omics, spatial transcriptomics, liquid biopsy, and patient-derived organoids may be integrated to delineate the evolutionary trajectory of PTX resistance and establish precision stratification systems based on molecular signatures, metabolic states, and tumor microenvironmental features. Ultimately, only through mechanism-guided, multi-target combinatorial interventions tailored to the dominant resistance drivers can the clinical benefit of anti-resistance therapies be meaningfully improved.
